# Impacts of Music Intervention on Dementia: A Review Using Meta-Narrative Method and Agenda for Future Research

**DOI:** 10.3390/neurolint13010001

**Published:** 2021-01-01

**Authors:** Mahsa Soufineyestani, Arshia Khan, Mina Sufineyestani

**Affiliations:** 1 Department of Computer Science, University of Minnesota Duluth, Duluth, MN 55804, USA; akhan@d.umn.edu; 2 The Urban Institute, School of Energy, Geoscience, Infrastructure and Society, Heriot-Watt University, Edinburgh EH14 4AS, Scotland, UK; ms316@hw.ac.uk

**Keywords:** dementia, music, music intervention, music therapy, clinical trial, and randomized controlled trials

## Abstract

Background: Dementia, a significant cognitive impairment, is characterized by a decline in memory. It affects an individual’s mood and behavior, which can impair their quality of life and well-being. Studies show that the demand for applying music as a new therapy method for dementia has increased during the last decades. Objective: To review the studies conducted on the impacts of music on different symptoms of dementia and provide readers with a more detailed picture of the efficacy of music, and recognize gaps in the literature. Methods: Electronic searches were conducted in the Cochrane Library (1938), Embase (773), Medline and PubMed (700), PsycINFO (89), and Scopus (218) databases. The search was comprised of all the literature from 2010 to 2020. For the search, key terms including “Dementia” AND “music” OR “music intervention” OR “music therapy” and index terms “clinical trial” OR “randomized controlled trials” were used. Finally, after screening 891 citations, 30 studies were included. Results: In general, it was observed that in most cases, music could be used as one of the safe and cost-effective non-pharmacological approaches for dementia treatment. However, in some studies, no impact or short-term effect of music on some symptoms of dementia such as wandering, agitation, and cognition was detected. Conclusion: Observing no effect or even negative impact of music on people living with dementia could be due to a random selection of music, fewer individuals, and the lack of a standard protocol. High heterogeneity in outcomes did not allow for clear conclusions on the benefits of music in dementia. This demands a comprehensive non-pharmacological music treatment approach to be designed for each stage of dementia to be employed alongside pharmacological treatments. This study proposes 13 gaps in the research on the health impact of music on dementia that could be studied by future researchers.

## 1. Introduction

Dementia is an umbrella term for several progressive diseases such as Alzheimer’s that affect memory, language, problem-solving, and the individual’s thinking ability, which interfere with their daily living activities [1]. People living with dementia (PWD) usually face social and communication interaction problems, which negatively impacts on their lives and the people around them [1].

There are various kinds of therapy methods for managing dementia symptoms: pharmacological and non-pharmacological. Although evidence shows that pharmacological therapy can delay and somewhat control behavioral disorders in PWD, it cannot cure dementia [2]. Some popular non-pharmacological therapies are pet therapy [3], robot therapy [4], reminiscence therapy [5], aromatherapy [6], occupational therapy [7], massage and touch therapy [8], doll therapy [9], light therapy [10], and creative arts therapies (music, dance-movement, and drama) [11]. Music interventions are categorized as ‘music medicine’ when individuals listen to pre-recorded music that is offered by medical personnel [12]. In contrast, music therapy is clinical and evidence-based use of music interventions to accomplish individualized goals within a therapeutic relationship by a credentialed professional who has completed an approved music therapy program [13]. Unlike drug therapy, applying music as a treatment approach usually does not have any side effects. For this reason, many physicians and caregivers promote and encourage music therapy, or music intervention as a beneficial and alternative treatment method for dementia [14]. Although using music as a treatment approach cannot cure dementia symptoms, it can reduce the symptoms [15]. Even with advanced dementia—when PWD have severe problems with judgment, planning, reasoning, speech, and language—their responses to music are undeniable, and its impact can last for hours or even days [16].

### Benefits of Music Treatment for People Living with Dementia 

Several physiological and psychosomatic benefits of music have been mentioned in the literature.

- **Biological/physiological:** The physiological effect of music helps to balance vital signs such as blood pressure, heart rate, respiratory rate, and hormone levels [17,18].
- **Behavioral and psychological**: The psychological effects of music help to reduce mood fluctuations and behavioral disorders such as depression, agitation, and aggression [2,19,20,21].
- **Cognitive:** Music also boosts communication skills, the quality of life, well-being, intimacy, memory, self and environmental awareness, the ability to distinguish between the surroundings and moments of the day, and managing pain [17,22,23]. Among all music advantages, some of the most important ones are enhancement in language skills, verbal and non-verbal expressions, improvement of social activity and communication, raising cognitive levels, and self-awareness [19,24]. PWD who have verbal communication problems can benefit from music as a non-verbal communication solution to express themselves without inhibitions.
- **Emotional:** Music engages the individual’s attention (apathy) and helps them enjoy their life, improves their self-esteem, and communication, which leads to improvement in behavior [25]. People living with dementia who listened to their favorite music were observed to have a decrease in stress level and an increase in relaxation and happiness [26].

**Figure 1:**
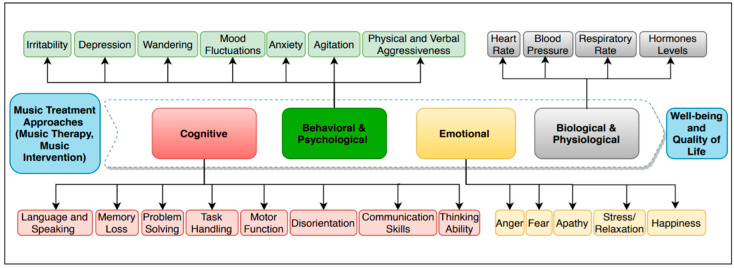
Benefits of music treatment for people living with dementia (PWD).

To retain the music benefits, people with dementia need to receive regular music treatment, which is approximately two to three times a week [27]. Overall, studies from the last 10 years have shown improvement in cognitive, emotional, and behavioral and psychological symptoms of PWD through music treatment approaches, resulting in a better quality of life and wellbeing (Figure 1).

Music intervention can be performed at home or in residential aged care facilities, as individual [28] or group therapy [29,30,31]. Group music therapy boosts communication and interaction skills between PWD and improves their relationships with their caregivers and family members [24]. Music intervention has two types: active or receptive [32]. In receptive mode, people living with dementia listen to the live, recorded, or background music for relaxation or changes in mood without any engagement [17,19]. On the other hand, active music therapy encourages the individual to be engaged with the music by singing along, playing basic musical instruments individually or within a group, moving their body to the rhythm, or dancing to the song. Singing can decrease behavioral disorders, improve mood, and enhance cognitive functioning. Singing also raises the heart rate and hormone levels [29,33]. Playing a musical instrument can prevent or postpone the onset of dementia symptoms [29,34]. Active music therapy improves PWD’s listening ability and helps them be aware of themselves, the environment, and the people around them. Researchers have sometimes used the term ‘passive’ instead of ‘perceptive’ for music medicine. Studies have shown that listening to music, specifically favorite and meaningful music, facilitates reminiscence and encourages more positive reactions. Listening to music can have calming impacts and help PWD to be connected to their family members, caregivers, and other residents [35]. 

Previous review studies have limited their focus to the following aspects:

- Specific symptoms of dementia such as depression [19,20,36,37], agitation [20,36,37,38,39], cognition and memory [2,14,40,41,42], quality of life/well-being [43,44], and anxiety [19,36,37,44];
- Group music therapy [45];
- Specific stage or type of dementia [20,41,46];
- Recorded music [21];
- Music and exercise [47];
- Assessment tools for measuring music’s impact [48];
- Residents living in a residential aged care facility [19]; and
- Individualized music [46].

The main aim of this review was to investigate music’s efficacy on people living with dementia by exploring the changes in the different symptoms of dementia including a broader setting. We also provide readers with a more detailed picture of the music’s efficacy by analyzing the gaps in previous studies and suggesting more appropriate research design and methodological considerations that need to be addressed in future studies.

## 2. Materials and Methods

### Search Strategy and Selection Criteria

**Figure 2:**
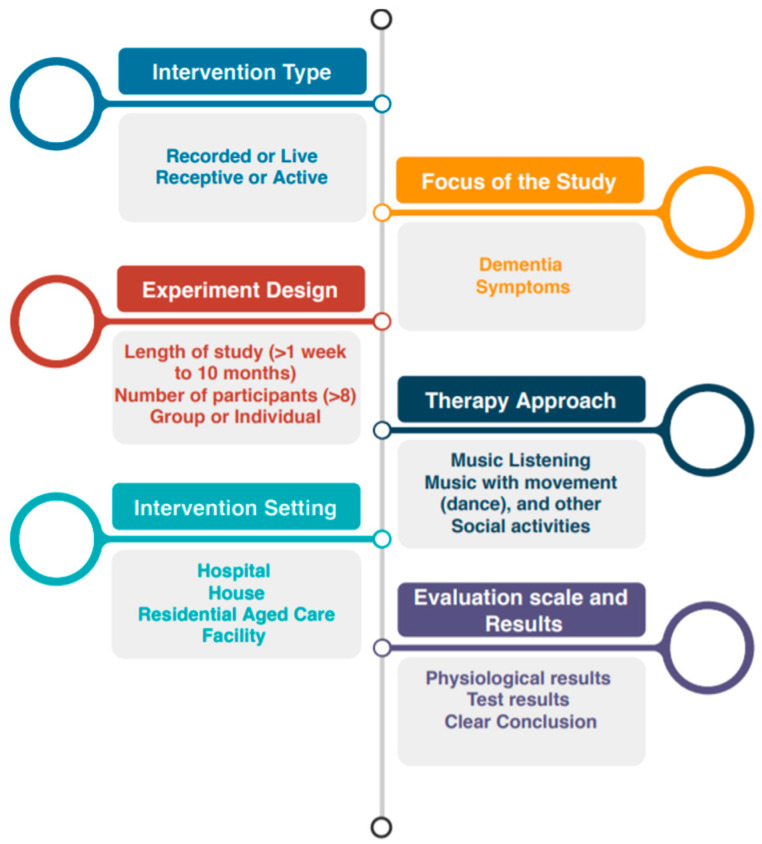
Inclusion criteria of studies.

The objective of this meta-narrative review was to investigate the publications using a qualitative approach and systematic review by highlighting the contrasts and similarity on music’s efficacy. Electronic searches in the Cochrane Library (1938), Embase (773), Medline and PubMed (700), PsycINFO (89), and Scopus (218) databases were performed to find all of the peer-reviewed publications in English that had the keywords and search terms “Dementia”, “music”, “music intervention”, “music therapy”, “clinical trial”, and “randomized controlled trials” in their titles and abstracts. The time frame chosen for this search was from 2010 to 2020. In general, studies that fit in all of the following criteria were included in this review:

- Intervention type
- The focus of the study
- Experiment design
- Therapy approach
- Intervention setting
- Evaluation scales and results (Figure 2)

Papers that did not have any details about one of the criteria as above-mentioned have not been included in this review. Additional articles were identified through a review of reference lists of included articles, titles, and abstracts. Identified citations were exported into Mendeley, and duplicates were removed.

## 3. Data Extraction and Quality Assessment

**Figure 3:**
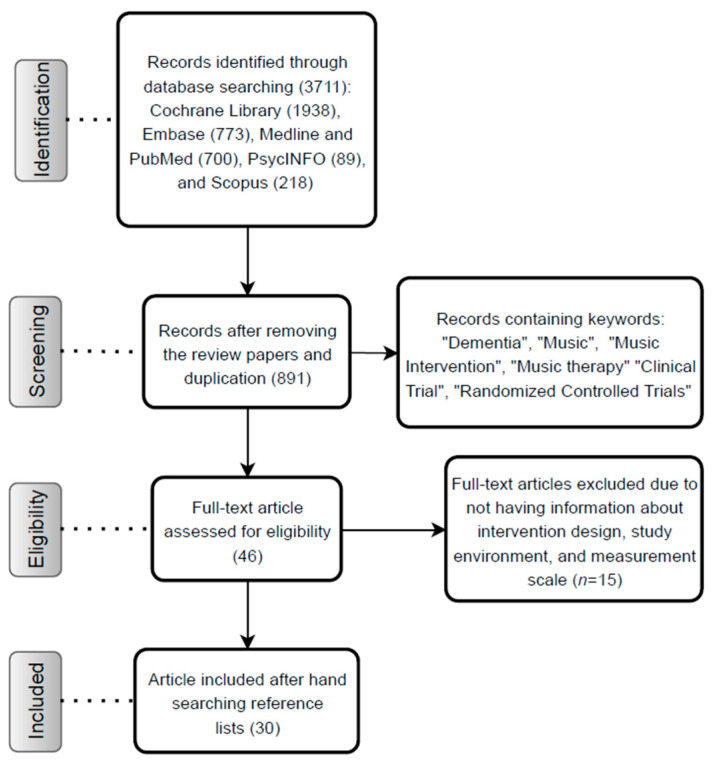
Flow diagram of this review study.

Among the 891 studies, only 30 met the inclusion criteria and were selected for further investigation (Figure 3).

## 4. Results

**Figure 4:**
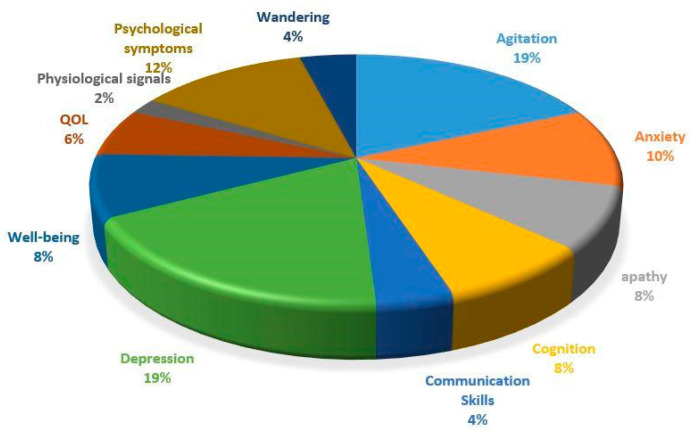
Percentage of music treatment research in each area of dementia.

We extracted data on the study areas, type of dementia, intervention design, number of participants, study environment, conductor of the study, measurement scales, and results. The measurement and results are summarized in the Appendix A. Table 1 and Figure 4 illustrate the percentage of the selected studies based on the common symptoms of dementia and the type of music intervention design. Among all of these studies, individual music intervention for people with dementia who lived in residential aged care facilities and listened to recorded music in a receptive mode has received more attention. Some researchers have undertaken a general investigation of physiological signals that include all of the related behavioral symptoms of dementia. However, it seems that depression, agitation, and anxiety have received more attention from researchers.

## 5. Gaps in the Research

Previous studies have identified some areas that need to be covered to draw clear conclusions on music’s efficacy for PWD. Some of the suggested areas such as combining MT with other activities [10,47,49], doing further research and methodological design [2,14,19,20,21,36,39,46,49], using a larger sample of randomized clinical trials [36,40,41,49,50], and considering the impact of music on different types and stages of dementia [49] are aligned with the items that we are going to discuss. Based on our investigation, the following gaps need to be addressed in experiment design and methodologies in future studies to have more reliable and evidence-based results.

- **Control group:** To compare individuals living with dementia who experience music treatment and others who only receive pharmacological treatment or are involved in other activities, it is necessary to divide people with dementia into two groups: a control group or a study group. Although sixteen papers out of 30 [18,29,30,34,38,50,51,52,53,54,55,56,57,58,59,60] included both groups in their studies, some studies did not have a control group in their experiments [21,26,28,33,61,62,63,64,65,66,67,68,69]. For example, in two studies [54,55] with both control and study groups, a greater behavioral disturbance was noticed between the study group than the control group. Additionally, in another paper [69] with only a study group, no changes was noted in memory. Thus, it is hard to conclude that music is an effective treatment for dementia, especially in the absence of a control group when there is a discrepancy between the results.
- **Gender:** Although researchers have included different genders (females and males) in their study, only one of the studies identified music’s impact based on gender [61]. In this study, individuals listened to unfamiliar childhood songs or a spoken version of visual stimuli, but no significant difference was observed between genders. Therefore, further cross-sectional studies are needed to determine if gender has any effect.
- **Sample size:** The sample size of the studies varied from eight to 165 participants. In several studies, the sample size of the experiment was less than ten [28,58,61,70]. For example, one research analyzed the behavior of nine PWD who received music therapy [28], where expressing more positive emotions and improvement in their well-being and communication level were observed. However, two studies [55,69] included a larger population (120 and 59, respectively) in their research, but did not find any significant changes in PWD symptoms. Hence, it is not clear that music caused changes in the level of dementia symptoms because of the use of a smaller sample size.
- **Long-term effect:** Some studies have found that music had a short-term impact, but it is unclear if music can be beneficial in the long-term. Only two studies reported long-term [28,34], one study reported short-term [50], and one study reported both short- and long-term effects of music [50]. In one study conducted in a residential age care facility [56], residents were divided into three groups doing activities such as music-movement, music listening, and social activities for 12 weeks. The residents’ agitation levels were monitored and compared before, during, and after involvement with the activities. Short-term change in the agitation level of those who were engaged with music-movement activity was noted. In contrast, no changes in agitation level of the residents in other groups were observed. Additionally, in another study conducted for 14 weeks, no statistically significant differences were observed in the memory of the residents [69]. As a result, more high-quality longitudinal studies are needed to monitor the benefits of music in dementia over a period of time, particularly in the long-term.
- **Live or recorded music:** Music intervention sessions are conducted either as a recorded [18,21,26,28,38,50,54,60,62,66,67,69] or live music [57] based on individual’s preference, or recorded [29,52,53,56,59,61,65] or live music [63] selected by caregivers, while some other studies have employed music therapy sessions either as a recorded music based on the individual’s preference [18,26], recorded [34,51,58,68], or live music [33,70] selected by music therapist. Five studies observed that listening to live music and being engaged with the singer and/or singing the song and/or playing musical instruments boosted the general well-being, mood, quality of life, and PWD’s relationships with others [33,57,63,67,70]. However, in some papers, changes in the health status of individuals living with dementia were observed while listening to recorded music [38,41,52,54,65], which implies no apparent differences in the reviewed studies between playing live or recorded music. Emotions connected to music might change the moods of people living with dementia. Further cross-sectional studies using a control group are required to determine if the music transferring method has any effect.
- **Music intervention or music therapy**: Researchers have sometimes mixed the definition of music intervention (music medicine) with music therapy and considered any types of music treatment as music therapy. Among the 30 reviewed studies, only nine of them considered music therapy [18,30,33,34,58,64,68,70] and the remaining investigated music intervention. Music therapy was examined either as an active therapy in individual [58,68,70] or group [30,33,34] modes, or receptive group therapy [18,64]. Music intervention is considered either as active [18,29,55,56,57,59,67], receptive [21,38,52,54,60,61,62,63,64,65,69], or both receptive and active modes [28,50]. Although most of the music therapy studies have reported positive changes in the individuals’ symptoms, in one study, no changes in wandering behavior were reported [33]. While more positive responses in emotional states, behavioral and psychological symptoms of dementia (BPSD), and cognition of individual’s living with dementia was reported using active and perceptive music intervention [28,50], researchers did not talk about the differences between active and perceptive modes. Studies that applied only one music transferring method had mixed results, and it is hard to conclude which approach worked better. More studies need to be conducted in order to understand which approach is more effective for dementia treatment.
- **Selecting appropriate music and professional therapists:** The music treatment approach aims to decrease the costs, and it is not obvious that it is necessary to hire professional music therapists to conduct music sessions, or medical personnel can conduct the therapy session. While some researchers have tried to use PWD’s favorite song [26,38,41,54,56,62,64] played by musicians, singers, music therapists, or trained caregivers (who are taught by a music therapist to conduct the music sessions) [18,33,50,54,57,58,61,63], others chose random songs performed by caregivers or facility staff [30,64,70]. This variation and inconsistency in the delivery of music make it hard to generalize the results. Therefore, further research on performing music with professional music therapists or facility staff is needed. Furthermore, perhaps music should be chosen based on the individual’s preference by asking their family members or conducting a survey to determine which type of music has a more restorative impact on PWD.
- **Physiological impact:** In addition to changes in psychological data, dementia may cause changes in physiological signals. One study [17] found that music is a beneficial modality to balance vital signs such as blood pressure, heart rate, and respiratory rate. Most of the studies focused on the psychological data and ignored physiological signals. Two studies used wearable sensors to measure physiological signals [58,71]. In one study [71], the impact of music therapy was examined on 12 elderly people with vascular dementia using an Electrocardiogram (ECG) sensor. They noted an increment in heart rate variability (HRV) features such as mean values of inter beat interval (RR), Root Mean Square of the Successive Differences (RMSSD), proportion of NN50 (pNN50), and high frequency band during the music therapy session and a decrease in values of HRV features after the sessions. The drawback of focusing on the statistical analysis of the observed or neurological data is that the collected data in these studies dealt with mostly subjective data rather than taking into consideration the physiological and psychological data that was gathered using wearable sensors, which can capture more accurate changes in physiological and psychological symptoms. Thus, it is impossible to reach an overall conclusion that music can improve PWD’s physiological signals.
- **Lack of an exact measurement scale:** Although most of the studies employed quantitative measurement mechanisms that used clinical scales, some researchers did not apply any particular measurement scale. Some researchers have monitored changes in PWD’s moods and behavior by observing and analyzing their body or facial expressions [41,57,65], or reviewed self-reported surveys or caregiver notes [53,63,70]. It is difficult to objectively assess the impact of complex multimodal intervention such as music. It seems that using some physiological measures such as heart rate, blood pressure, skin conductance, measuring stress hormones, and analyzing the brain signals would be beneficial to provide us with more reliable measures.
- **Combination of music with pharmacological methods:** One study [20] pointed out that applying non-pharmacological treatments like music intervention or therapy and pharmacological treatment could mitigate symptoms of anxiety and depression in people with mild dementia, while it is unclear that observing all the changes in individual situations was only due to the addition of music to their treatment or combining music with pharmacological solutions. Therefore, further studies with a control group are necessary to explore whether control variables such as pharmacological treatments affect dementia symptoms.
- **Combination of music with other activities:** Although some studies have proved that listening to music and being involved with social activities positively impact dementia symptoms, only nine studies combined various activities such as playing games, solving word-puzzles, gardening, and engaging in mental or physical activities [18,29,34,52,56,57,59,60,68]. These studies have shown that combining social activities with music intervention or therapy can improve or postpone dementia symptoms. Additionally, individuals who engaged in social activities demonstrated improvement in communication skills [22]. Several researchers applied music and playing games to reduce agitation, aggression, apathy, and anxiety levels, enhance communication skills, and improve emotional expressions [34]. In another study [60], PWD were divided into three groups engaged in either social activity, listening to music, or music with dance. It was concluded that the combination of music with dance could improve cognitive function, memory, and depressive symptoms while there was no significant changes in agitation among the three groups. In one study [18], short-term reduction in agitation behaviors of PWD was reported while doing either social activities or listen to music played by a music therapist. Researchers incorporated 77 PWD in their study and compared changes in their behavior while listening to the music, singing along to the song and dancing, or doing daily recreational activities such as handwork, solving a puzzle, and cooking designed by the occupational therapists. These outcomes indicated that keeping PWD occupied with functional tasks might help with declining dementia symptoms. These studies are useful, but since they do not study music treatment approach in isolation, it is not apparent that music or other social activities improve their psychological symptoms. To have more reliable and valid results, there should be more studies examining the combined effect of music with other activities involving individuals in both activities during a period of time and observing their behaviors for each type of activity.
- **Impact on agitation, wandering, and cognition:** The impact of music treatment on the agitation, wandering, and cognition of individuals with moderate or severe dementia is not completely clear. While seven out of 30 studies discussed a reduction in agitation [29,33,34,52,55,57,65], others reported no change in agitation. For example, in one study, no changes were noticed on wandering and agitation [33]. Another study [59] did not find any differences in the individuals’ cognition levels. Thus, more studies are required to investigate the impacts of music on agitation, wandering, and cognition.
- **Benefits of music for family members and caregivers:** In one study, it was mentioned that music therapy could also increase caregivers’ satisfaction [26]. The impact of music was examined on eight PWD and their caregivers while professional music therapists taught caregivers to play PWD‘s favorite music by themselves at home. The comparison between the data revealed a decrease in the stress level and an increase in both groups’ relaxation and happiness. However, more studies like these should be conducted to determine music’s impact on family members and caregivers.

## 6. Discussion and Conclusions

This meta-narrative review on the previous studies indicated some improvement in the physiological or psychosomatic behaviors of people living with dementia after music intervention [30,34,38,51,57,67,70]. For example, music intervention may cause decrease in agitation [2,19,21,36,38,39,50], anxiety [19,36,37,43,45,50], depression [19,37,43,49,50], behavioral and psychological symptoms [2,43,44,72], and boost cognition, memory [2,14,19,37,41,44,46,48,49], motor outcome, and quality of life [19,43,44,49], while there are studies that did not reach any clear conclusion about the effectiveness of music [18,62]. For instance, two studies showed no evidence or significant enhancement in the behavior and mood of people living with dementia [33,55]. These reviewed studies gave general results that make it hard to generalize conclusions regarding the efficacy of music intervention in dementia care. Therefore, this inconsistency between the results of music on agitation, wandering, and cognition demands further research in these areas. These results are in line with those previous systematic and meta-analysis reviews of the music’s impact on PWD that reported little or no effect on cognition [2,19,26,40,41,44,46,73], agitation [19,37,38,39,44], depression [19,20,37,44,47], anxiety [19,20,37,44,45,47], aggressive behaviors [19], and quality of life [19]. For example, in one meta-analysis study [20] on music’s effect on cognition, it mentioned that music therapy might be a complementary treatment if its impact is considered on a larger sample of randomized control trials [40]. Another problem with dementia is a sense of apathy; one solution to this could be involving individuals’ with different activities. For example, a combination of music intervention with other activities such as occupational therapy, social engagement, and pharmacotherapy may help to retain memory and decrease agitation behaviors. Another study [14] of cognitive function concluded that combining music therapy with cognitively stimulating activities such as dance, physical exercise, video game, and art can caused reduction in cognitive decline. The reviewers suggested drawing a more reliable conclusion required more evidence and a rigorous methodological investigation [14]. Another meta-analysis on the combination of music and physical exercise [74] showed that the rhythmical music that involved PWD could be beneficial for some individuals. A research on the music’s efficacy on the anxiety level of individuals with mild to server dementia [45] reported a decline in anxiety and suggested further research by considering larger group size, different range of age, and standardization of the best time for treatment [45].

Overall, these studies have focused on the short-term impacts of music on people with dementia. One of the drawbacks with the previous studies is using the terms incorrectly in the literature and referring to any type of musical intervention as a music therapy. Thus, it is important to distinguish between using music intervention and music therapy. Music intervention or therapy has mixed outcomes, which do not guarantee it as a long-term therapy solution. Therefore, to have more reliable results, high-quality longitudinal, cross-sectional studies should be conducted to identify the confounding factors. Additionally, a bigger sample size with both control and study groups is needed. Additionally, controlling for pharmacological therapy and other intervention methods could be beneficial for investigation into the impact of music in isolation and also in combination with other treatments. Additionally, researchers should examine whether recorded music can have the same outcome as live music, which would decrease the cost for hospitals and nursing facilities. Fewer studies on personalized music intervention [21,54,57] demands more studies based on the culture, age, gender, dementia stage, type of dementia, and availability of treatment resources.

One of the symptoms of dementia is changes in vital signs such as blood pressure, heart rate, and vitamin deficiency. This review found three papers that considered vital signs [17,58,71] given that music can stabilize blood pressure and heart rate, improve appetite, sleep, and quality of life, which would be a valuable cost effective intervention. Thus, more research is needed to understand if it is necessary to use various measurement scales including physiological and psychological data to track changes in the individuals’ symptoms.

Additionally, only two papers considered the benefits of music for family members and caregivers [66,68], which need more studies. This will help to determine if music has any restorative impact on family members and caregivers.

Overall, for future research, it is beneficial to consider all aspects of the methodological considerations discussed in this review including gender, control group, sample size, long-term effects, whether it is live or recorded, receptive or active music intervention or therapy, personalized or selected music by caregivers or music therapists. Furthermore, combining music treatment with other activities that involves both PWD and their family or caregivers may be beneficial, along with targeted pharmacological treatments.

## Figures and Tables

**Table 1:** Percentage of experiment design based on intervention type, setting, and dementia type music intervention (MI)/music medicine, and music therapy (MT).

Type of Cognitive Disorder	Intervention Type	Individual/Group Therapy	Intervention Setting
Dementia (all type): 83.4% Alzheimer’s only: 3.3% Dementia (all types) & Alzheimer’s: 13.3%	Active MI: 26.6% Receptive MI: 36.6% Receptive & Active MI: 6.7% Active MT: 13.3% Receptive MT: 16.7% Live: 10% Recorded: 90%	Group: 30% Individual: 70%	Family home: 6.7% Hospital: 6.7% Residential aged care facility: 86.6%

## Appendix A

**Table A1:** Summary of studies on music intervention for dementia-related diseases.

Author	Outcome Focus Area	Dementia Alzheimer’s	Intervention Details & Measurement Frequency	No. Participants Control Group (CG)/ Music Therapy (MT)	Study Environment & Conductor	Measurement Method/Results
Moore, 2010 [52]	Apathy, agitation, and appetite	Early to severe Dementia	3 weeks (25 min, 30 min before lunchtime), twice a week) Recorded familiar music or physical activity (Seated chair exercise and music listening) Music intervention Pre, Post data	84 (MT and CG)	Residential aged care facility Research assistant	Measurement scales: Frontal systems behavior scale, Cohen–Mansfield Agitation Inventory (CMAI) Results: Significant changes in apathy and dietary intake but no change in agitation or eating ability
Raglio et al., 2010 [51]	Behavioral Symptoms	Severe Dementia	4 weeks (3 sessions in a month, for 30 min, One-month washout) Music therapy Group MT (3 people in a group)	60 (30: MT, 30: CG)	Residential aged care facility Facility staffs	Measurement scales: Mini-Mental State Examination (MMSE), Barthel Index and Neuropsychiatry Inventory (NPI) Results: A decline in behavioral disorder, improve in delusion, agitation, and apathy
Sung et al., 2010 [38]	Anxiety	Dementia	6 weeks (30 min twice a week) Recorded preferred music Music intervention Pre, End of each week data	52 (29: MT, 23: CG)	Residential aged care facility Facility staffs	Measurement scale: Rating Anxiety in Dementia (RAID) Result: Lower levels of anxiety
Cook et al., 2010 [57]	QOL and Depression	Dementia	12 weeks (30 min for 3 times in a week) Active, personalized, live music, Reading activity Music intervention Pre, During, Post data	47 (reading and music groups)	Residential aged care facility Musicians	Measurement scales: Evaluation of Dementia Quality Life (DQOL), Geriatric Depression Scale (GDS) Results: A higher level of midpoint QOL, increase in self-esteem and decrease in depression level
Lin et al., 2010 [29]	Agitation	Dementia	12 MT(Twice a week, 30 min MT for 6 weeks) Group MT, active (rhythmical music and slow-tempo instrumental activities, singing, listening, glockenspiel, musical activities and traditional holidays, music creator) Music intervention Pre, Middle, End, one month after MT data	100 (49: MT, 51: CG)	Residential aged care facility Facility staffs	Measurement scales: CMAI, Generalized Estimating Equations (GEFs) Results: Reduction in agitation and physical aggressive behavior after beginning MT, reduction in verbal aggressive behaviors only in the middle of MT
Stern et al., 2010 [61]	Recognition Ability	Alzheimer’s	30 min Unfamiliar childhood songs, song lyric or spoken version visual stimuli Music intervention During data	27 (13: Alzheimer’s, 14: Healthy)	Residential aged care Trained Musician	Measurement scales: ANOVA memory test Results: No differences between genders, healthy individuals had better recognition ability than patients with Alzheimer’s
Ho et al., 2011 [65]	Behavior	Dementia	4 weeks (Twice a day, during meal time) Recorded calming music Music intervention Pre, Post	22 (M: 10, F: 12)	Hospital-based residential aged care facility Researcher	Measurement scale: CMAI Result: Decline in agitation
Nair et al., 2011 [53]	Physical aggressiveness, Verbal abuse, Agitation, wandering and Inappropriate sexual advances	Dementia	4 weeks (for 4 h) Recorded and Baroque Music Music intervention Pre (2 weeks), During (4 weeks), Post (2 weeks)	75 (MT and CG)	Residential aged care facility Facility staffs	Measurement scale: Behavior observation Results: Listening to Baroque music has a negative effect on patient behaviors, a more behavioral disturbance was observed between experimental groups than the control group.
Hanser et al., 2011 [26]	Mood and Psychological State	Dementia	8–20 sessions Favorite music Music therapy Pre, During, Post data	8	Residential aged care facility Caregiver	Measurement scale: Visual Analog Scale (VAS) Results: Both patients and caregivers have a higher level of relaxation, happiness, and comfort after MT
Terworth & Probst, 2011 [34]	Behavioral and Psychological Symptoms	Mild to Moderate Dementia	6 months Group MT (6–10 patients), active(singing, playing an elementary musical instrument, listening to biography of music and Playing game (word-association, puzzle)) Music therapy Pre, Post data	49 (MT: 26 (M: 3, F: 23), CG: 23 (M: 3, F: 20))	Residential aged care facility Caregivers	Measurement scales: Mini-Mental Status Test (MMST), Global Deterioration Scale Neuropsychiatric Inventory (NPI), GDS, Inventory to Assess Communication, Emotional Expression and Activity in Dementia (ICEA-D) Results: Reduction in agitation, aggression, apathy, and anxiety beside an improvement in communication, emotional expression
Janata, 2012 [54]	Agitation and Depression	Moderate and Severe Dementia	12 weeks (3 h for several times in a day) Recorded and Customized music (Widespread and frequent personalized music) Music intervention Pre, Post data	38 (25: F, 13: M,19: MT, 19: CG)	Residential aged care facility Music therapist	Measurement scales: CMAI, NPI, Cornell Scale for Depression in Dementia (CSDD), MMSE Results: Reduction in agitation and depression level of patients
Vink et al., 2012 [18]	Agitation	Dementia	4 months (40 min twice a week) Group MT, favorite music or recreational activities Music therapy Daily, Post data	77 (43: MT, 34: Recreational activities)	Residential aged care facility Trained music therapist	Measurement scales: CMAI Results: Decline in agitation level during listening to music but after music intervention agitation comes back
Vleuten et al., 2012 [63]	Quality of life, Communication skills and mental well-being	Mild and Severe Dementia	45 min (one or a few songs) Intimate live music Group therapy (10 patients in each group) Music intervention Post data	45	Residential aged care facility Professional singers	Measurement scales: Behavior observation Results: Improvement in human contact, communication skills, observing more positive emotions and less negative emotion
Baker et al., 2012 [66]	Anxiety, Depression, Spousal Relationship	Dementia	6 weeks (20–30 min 3 sessions per week) Active, familiar/preferred/quiet music Music intervention Pre, post data	5 couples (M: 2, F: 3)	Home Caregivers	Measurement scales: GDS, Geriatric Anxiety Inventory (GAI), Mutual Communal Behaviors Scale (MCBS), Positive Aspects of Caregiving Questionnaire (PACQ), NPI Results: Improve spouse relationship, satisfaction, enjoyment, and well-being of caregiver, and boost the mood of couples
Sakamoto et al., 2013 [50]	Behavioral and Psychological Symptoms	Advanced Dementia	10 weeks (30 min for once a week) Passive/active, preferred music Music intervention Pre, During, Post data	39 (3 groups: CG (F: 11, M: 2), passive (F:10, M:3), active (F:11, M:2))	Residential aged care and Dementia hospital Music facilitator	Measurement scales: Nerve Index and Faces Scale behavioral, Behavioral Pathology in Alzheimer’s Disease (BEHAVE-AD), Heart rate (HR) Results: Improve in emotional states (Both short and long term), more improve in BPSD among active MT group
Park, 2013 [62]	Agitation	Dementia	4 session (30 min twice a week before peeking of agitation) Favorite music Music intervention Pre, During, Post data	26	Residential aged care facility Facility staffs	Measurement scale: CMAI Results: Decline in agitation level while listening to music but after music intervention agitation comes back
Gold, 2014 [70]	Mood and Behavior	Advanced Dementia	4 months (once in a week) Active, Live music Music therapy 4 days after each MT session, End of MT sessions	9	Hospital Researcher	Measurement scales: Caregiver notes (observation) Result: 8 out of 9 patients showed positive changes
Chu et al., 2014 [30]	Depression and Cognition	Dementia	12 sessions (30 min twice a week) Group therapy, active Music therapy Pre, Middle, End, Post data	104 (MT, CG)	Residential aged care facility Facility staffs	Measurement scales: Chinese Version of C-CSDD and Salivary Cortisol, The Chinese version of the MMSE for measuring cognitive function Results: Reduction in depression level, improvement in cognitive function
Eggert et al., 2015 [64]	Behavioral Changes	Alzheimer’s Dementia	image and music: 1–4 weeks, wash out:4 weeks, image and music:4 weeks (1.5 h per week) Group therapy Music intervention Pre, Post data	24	Residential aged care facility Facility staffs	Measurement scales: Individualize Dementia Engagement and Activities Scale tool, Montreal Cognitive Assessment, CMAI Results: Reducing the behavioral disorder
Raglio et al., 2014 [17]	Behavioral and Psychological Symptoms (Depression, Anxiety, Apathy, and Cognitive)	Moderate to Severe Dementia	10 week (20 music 30 min twice a week) Active, individualized music Music intervention Pre, Post data	120 (3 groups, CG, MT, and Individualized listening to music)	Residential aged care facility Facility staffs	Measurement scales: NPI, CSDD Results: No significant changes in behavioral and psychological symptoms
Schall et al., 2015 [28]	communication behavior and emotional well-being	Advanced Dementia	20 sessions for 6 months (3 cycles of music for 23–39 min) Active/passive, individual video graphed music Music intervention Pre, During, Post data	9	Home Caregivers	Measurement scales: NPI, The CODEM instrument for assessing communication behavior, The Positive Response Schedule for Severe Dementia (PRS) for assessing well-being, The Observed Emotion Rating Scale (OERS) for rating positive and negative emotions, Results: Improvement in communication skills, well-being, and expressing more positive emotions
Hsu et al., 2015 [58]	Well-being Dementia symptoms	Dementia	5 months (30 min music once a week) Active, well-known songs Music therapy Pre, During, End, Post data	17 (MT, CG)	Residential aged care facility Music therapist	Measurement scales: NPI for Nursing Homes for measuring Dementia symptoms, Dementia Care Mapping (DCM) for the well-being Results: Physiological data heart rate and skin conductance, skin temperature and bodily acceleration, Decrease in NPI for MT and increase in NPI for CG after 5 months, Improvement in the well-being of MT and decline in the control group, Improve in the interaction between patients and caregivers
Ray and Mittelman, 2017 [33]	Agitation, wandering and Depression	Moderate and severe Dementia	15 min–60 min Preferred music, live, active group therapy (4–6 patients) Music therapy Pre, During, Post data (each for 2 weeks)	132 (F: 112, M: 20)	Residential aged care facility Music therapist	Measurement scales: ANOVA Results: A decrease in agitation and depression, no change in wandering
Melhuish et al., 2017 [68]	QOL	Semantic and frontotemporal Dementia	50–60 min once a week Active (music from the 1920s to 1960s), Dance/Movement Music therapy	24 (M:12, F:18) (15: Moderate Dementia, 12: Advanced Dementia)	Residential aged care facility caregivers	Measurement scales: Interpretative phenomenological analysis (IPA) Results: Help caregivers to discover patients’ skills and feeling, improve the connection between caregivers and patients
Tang et al., 2018 [59]	Apathy, Cognition	Dementia	12 weeks (50 min for 3 times in a week) Active, group therapy, playing a musical instrument, nostalgic music (nostalgic red songs, nostalgic nursery rhymes, and nostalgic Cantonese opera) Music intervention	77 (M: 39, F: 38, 39:CG, 38: MT)	Residential aged care facility Research assistant	Measurement scales: Apathy Evaluation Scale (AES), Mini-Mental State Examination (MMSE) Results: Decrease in apathy, no changes in cognition
Garrido et al., 2018 [21]	Psychological and Behavioral Symptoms (Depression, Anxiety, Apathy, and Cognitive)	Dementia	~30 min (2 min baseline 1–2 playlist (contain 2–4 song) each 8–9 min, 2–3 min between each playlist) Recorded personalized/preferred music (belong to 1930s–1970s) Music intervention Pre, During data	99	Residential aged care facility Facility staffs	Measurement scales: Activation of facial action (webcam Observed Emotion Rating), OERC Results: People with high levels of depression and with symptoms of Alzheimer’s type of Dementia demonstrated increased levels of sadness, People with low depression but high levels of apathy demonstrated the highest behavioral evidence of pleasure during music listening, although behavioral evidence declined with the severity of cognitive impairment
Cheung et al., 2018 [56]	Cognitive Functions, Depression, and Anxiety	Moderate Dementia	12 weeks 3 activities (music-with-movement(MM), intervention music listening(IML), and social activity(SA)) Music intervention Pre, Middle, End data	165 (3 groups; MM: 54, IML: 58, SA: 53)	Residential aged care facility Facility staffs	Measurement scales: Mixed multivariate analysis of variance (MANOVA), RAID scale for measuring anxiety, GDS, MMSE, Fuld’s Object Memory Evaluation (FOME), Modified Fuld Verbal Fluency Test (MVFT), Digit Span Test (DST) for measuring adult intelligence Results: Improvements in memory and depressive symptoms
Gulliver et al., 2019 [67]	Well-being, QOL, Mental Health (Depression)	Alzheimer’s Dementia	40–60 min for 8 weeks Music engagement program based on patients age, culture, and preference Music engagement program Pre, Post data	19	Residential aged care facility Facility staffs	Measurement scales: Visual Analogue Survey (VAS) based on WONCA diagram for Feeling and Social Activities, Cornell Scale for Depression in Dementia and quality of life through measuring factors of mood-related signs Result: Improvement in well-being and mental health
Cheung et al., 2020 [60]	Agitation	Moderate Dementia	6 weeks (45 min twice a week) 3 activities (music with movement(MM), music listening(ML), social activity(SA)), preferred music Music intervention Pre, Post data	165 (3 groups: MM, ML, SA)	Residential aged care facility Facility staffs	Measurement scales: A Chinese version of the CMAI Results: No statistically significant changes in agitation among the three groups, only short-term impact during the MT
Kwak et al., 2020 [69]	Agitation, Cognition	Dementia/Alzheimer’s	14 weeks Preferred recorded music Music intervention Pre, During, and Post data	59	Residential aged care facility Facility staffs	Measurement scales: CMAI, NPI-NH Result: No statistically significant changes in memory
